# Effect of citrus-based products on urine profile: A systematic review and meta-analysis

**DOI:** 10.12688/f1000research.10976.1

**Published:** 2017-03-06

**Authors:** Fakhri Rahman, Ponco Birowo, Indah S. Widyahening, Nur Rasyid

**Affiliations:** 1 Department of Urology, Dr. Cipto Mangunkusumo National General Hospital, Faculty of Medicine, Universitas Indonesia, Jakarta Pusat, 10430, Indonesia; 2 Department of Community Medicine, Faculty of Medicine, Universitas Indonesia, Jakarta Pusat, 10310, Indonesia; 3 Centre for Clinical Epidemiology & Evidence-based Medicine, Dr. Cipto Mangunkusumo National General Hospital - Faculty of Medicine, Universitas Indonesia, Jakarta Pusat, 10430, Indonesia

**Keywords:** Citrus, citrate, potassium citrate, urolithiasis, urine profile

## Abstract

***Background*** *. *Urolithiasis is a disease with high recurrence rate, 30-50% within 5 years. The aim of the present study was to learn the effects of citrus-based products on the urine profile in healthy persons and people with urolithiasis compared to control diet and potassium citrate. ***Methods.*** A systematic review was performed, which included interventional, prospective observational and retrospective studies, comparing citrus-based therapy with standard diet therapy, mineral water, or potassium citrate. A literature search was conducted using PUBMED, COCHRANE, and Google Scholar with “citrus or lemonade or orange or grapefruit or lime or juice” and “urolithiasis” as search terms. For statistical analysis, a fixed-effects model was conducted when p > 0.05, and random-effects model was conducted when p < 0.05. ***Results.*** In total, 135 citations were found through database searching with 10 studies found to be consistent with our selection criteria. However, only 8 studies were included in quantitative analysis, due to data availability. The present study showed a higher increased in urine pH for citrus-based products (mean difference, 0.16; 95% CI 0.01-0.32) and urinary citrate (mean difference, 124.49; 95% CI 80.24-168.74) compared with a control group. However, no differences were found in urine volume, urinary calcium, urinary oxalate, and urinary uric acid. From subgroup analysis, we found that citrus-based products consistently increased urinary citrate level higher than controls in both healthy and urolithiasis populations. Furthermore, there was lower urinary calcium level among people with urolithiasis. ***Conclusions. ***Citrus-based products could increase urinary citrate level significantly higher than control. These results should encourage further research to explore citrus-based products as a urolithiasis treatment.

## Introduction

Humans have suffered urinary tract stones for centuries ^1^. The incidence and prevalence of urolithiasis are different between geographic locations, depending on age and sex distribution, stone composition and stone location ^2^. Risk of stone development has been shown to be 5–10% with a higher prevalence in men than women ^3^. Urolithiasis is a common disease with significant morbidity and cost worldwide ^4– 6^. Based on National Health and Nutrition Examination survey, kidney stones affect 1 in 11 people in the United States, and an epidemiological increase was found in 2012 compared to 1994 ^7^. Additional data from Dr. Cipto Mangunkusumo National General Hospital, Indonesia’s national referral hospital, showed an increase in stone disease prevalence from 182 patients in 1997 to 847 patients in 2002 ^8^. Moreover, it is further worsen by its high recurrence rate reaching 30–50% within 5 years ^7^.

Calcium-based urinary tract stone is the most common stone composition found in urolithiasis ^9, 10^. Supersaturation is believed to be the mechanism behind calcium stone formation ^11^. One factor in determining urine stone formation or stone recurrence is urine profile, which is defined as urine volume and its composition. Hypercalciuria and hypocitraturia are the most common urine abnormalities found among calcium stone-formers ^12^. A high fluid intake could prevent stone formation by lowering supersaturation, whereas citrate could prevent stone formation by ionizing urinary calcium ^13, 14^. Food that is rich of citrate is citrus. There are wide variety of citrus fruits and derivate products that can be easily obtained, such as lemonade, grapefruit, orange, lime, and citrus-based juice. Several studies had already been conducted to learn the effect of citrus-based products on urine profile. However, the results between those studies were contradictive. Therefore, our study aimed to systematically review and quantify the available studies regarding the effects of citrus-based products on urine profile and its comparison to a control diet and potassium citrate.

## Methods

### Eligibility criteria

We included both healthy people and patients with urolithiasis history in our selection criteria. Study subjects must have consumed citrus fruits, such as orange, lime, grapefruit, or juices made from the fruits. Study designs could be interventional, prospective observational, or retrospective with standard diet therapy (any kind of mineral water), or potassium citrate, as a control group therapy. We included studies with urine profile as the outcome. We only included articles written in English or Indonesian, and those with full text article available. We excluded non-systematic review articles. We did not limit studies based on their year conducted.

### Search strategy

A literature search was conducted using PUBMED, COCHRANE, and Google Scholar as search engines on August 2016. The terms “citrus OR lemonade OR orange OR grapefruit OR lime OR juice” AND “urolithiasis” were used as search terms. We also searched the list of references in included studies. We did not use any limitation in study searching.

### Study selection and data extraction

All studies were screened for duplication using EndNote X6 software. Duplication-free articles underwent title and abstract examination using predetermined inclusion and exclusion criteria mentioned above. Selection of studies was selected by two authors independently. Discrepancies of opinion were resolved by discussion. All studies, which fulfilled the inclusion and exclusion criteria, underwent full text review. For every eligible full text, we extracted the following data, if available: subjects specific condition, citrus-based product used in the study, number of patients consuming citrus-based product, citrate content or its concentration, control intervention, number of individuals under control intervention. For the outcomes, we extracted urine profile data as follows: volume, pH, calcium level, citrate level, oxalate level, and uric acid level. Measurement units used in this study are L/day for urine volume and mg/day for urinary calcium level, urinary citrate level, urinary oxalate level, and urinary uric acid level. All data in the form of numbers were extracted manually as mean and standard deviation for variable measurement.

### Assessment of bias and statistical methods

This study used Cochrane Risk of Bias assessment tools ^15^ and Newcastle-Ottawa scale ^16^ to assess interventional and retrospective study’s quality, respectively. These assessments of study quality were done by two authors independently. Quantitative synthesis of included studies was analyzed using Review Manager (RevMan) 5.0 software and mean difference was used as its effects size measurement. Heterogeneity of studies was assessed using chi-square. A fixed-effects model was conducted when p > 0.05, whereas a random-effect model is conducted when p < 0.05. We also conducted subgroup analysis to differentiate between healthy and urolithiasis populations.

Studies which could not be included in quantitative analysis were described qualitatively.

## Results

**Figure 1.**
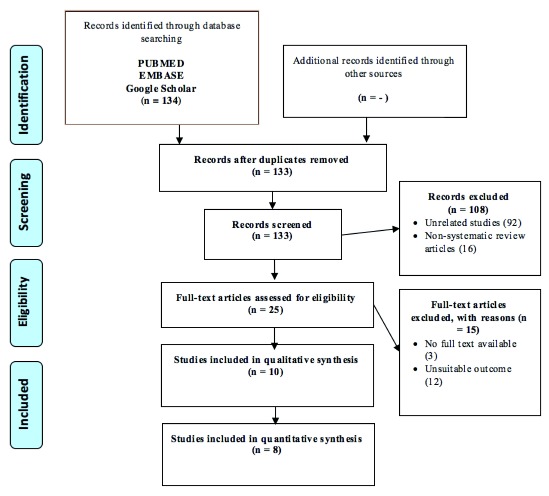
Study flow diagram.

We found 135 citations through database searching. Literature searching from the list of references found similar studies that were all already included in this study. Ten studies were found to be consistent with our selection criteria ( Figure 1).

**Figure 2.**
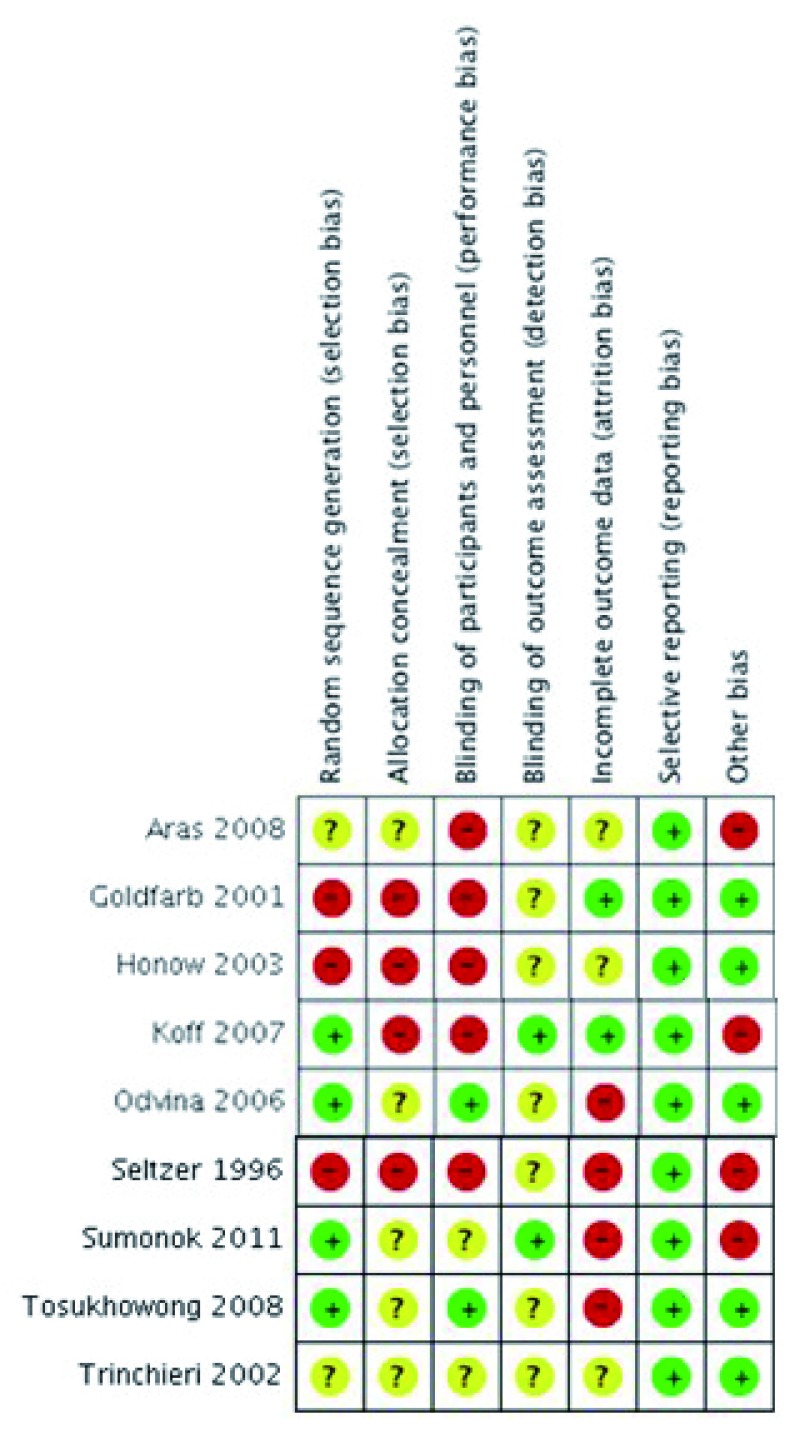
Risk of bias assessment summary.

Two of ten studies had to be excluded from quantitative analysis because of the following reasons: (1) Penniston *et al.* ^17^ only published baseline data and its maximal change following intervention; and (2) Tosukhowong *et al.* ^19^ used medians as their outcome measurement, and due to its non-uniform distribution, we were unable to convert these to means. Therefore, eight studies were analyzed to find the effect of citrus-based products on urine profile compared to controls. However, not all of the eight studies were included in urine profile outcome measurement, due to data availability. Characteristics of the included studies and their risk of bias assessment can be seen in Table 1 and Figure 2/ Supplementary Table 1, respectively.

**Table 1.** Characteristic of included studies.

Study author and year	Type of study	Subject condition	Intervention	n	Control	n
***Study included in qualitative synthesis only***						
Penniston *et al* (2007) ^17^	RS	Subjects with calcium oxalate stone	Lemonade (5.9 gr citrate)	63	Lemonade (5.9 gr citric acid) plus potassium citrate	37
Tosukhowong *et al* (2008) ^19^	RCT	Post-operative subjects with nephrolithiasis	• Lime powder (4.4 gr citrate) • Potassium citrate	13 11	Placebo	7
***Study included in qualitative and quantitative synthesis***						
Aras *et al* (2008) ^20^*	RCT	Subjects with hypocitraturic calcium stone	• Lemon juice (4.2 gr citrate) • Potassium citrate	10 10	Water 3 L/day	10
Goldfarb and Asplin (2001) ^21^	CBAS	Healthy subjects	Grapefruit juice	10	Tap water 240 ml, 3 times a day	10
Honow *et al* (2003) ^22^	CBAS	Healthy subjects	• Orange juice • Grapefruit juice • Apple juice	3 3 3	Mineral water	3
Koff *et al* (2007) ^23^	CS	Subjects with history of nephrolithiasis	Lemon juice (4.5 gr citrate)	21	Fluid except lemonade or citrus drink	21
Odvina (2006) ^24^	CS	Healthy and stone former subjects	• Orange juice (2.3 gr citrate) • Lemonade (2.3 gr citrate)	14 14	Distilled water 400 ml	14
Seltzer *et al* (1996) ^25^	CBAS	History of hypocitraturic calcium nephrolithiasis	• Lemonade (5.9 gr citrate)	12	Fluid maintaining 2 L urine	12
Sumorok *et al* (2011) ^18^	CS	Healthy subjects	• Sunkist orange soda (3 cans)	12	Water 1.06 L/day	12
Trinchieri *et al* (2002) ^26^	CS	Healthy subjects	• Grapefruit juice (1.4 gr citrate)	7	Water	7

RS – retrospective study; RCT – randomized controlled trial; CBAS – controlled before-after study; CS – crossover study. *Also included in qualitative synthesis for comparison between citrus-based product and potassium citrate.

### Effect of citrus-based products on urine profile

**Figure 3.**
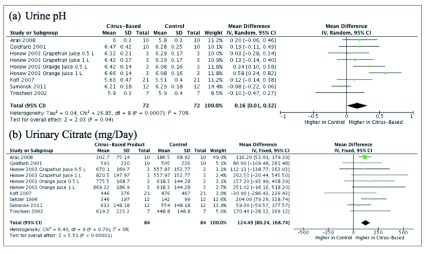
Urine pH and urinary citrate levels represented by a forest plot.

Data shows that citrus-based products increased urine pH (mean difference, 0.16; 95% confidence interval [CI] 0.01-0.32) and urinary citrate (mean difference 124.49; 95% CI 80.24-168.74) to a higher extent than control treatment ( Figure 3).

**Figure 4.**
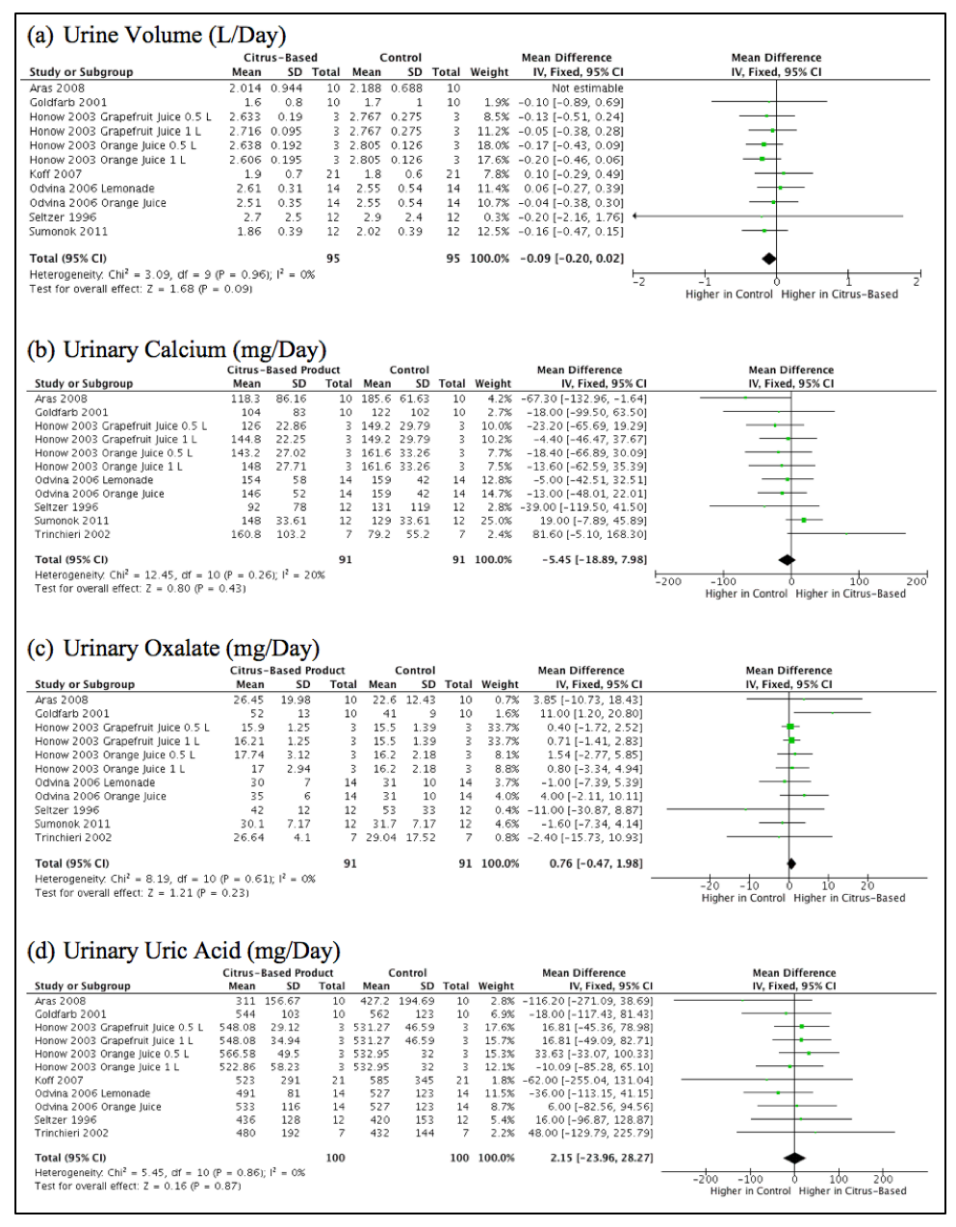
Urine volume, urinary calcium, urinary oxalate, and urinary uric acid levels represented by a forest plot.

However, there was no statistically significant difference in urine volume (mean difference -0.09; 95% CI -0.20-0.02), urinary calcium (mean difference -5.45; 95% CI -18.89-7.98), urinary oxalate (mean difference 0.76; 95% CI -0.47-1.98) and urinary uric acid (mean difference 2.15; 95% CI -23.96-28.27) between the two groups ( Figure 4).

**Figure 5.**
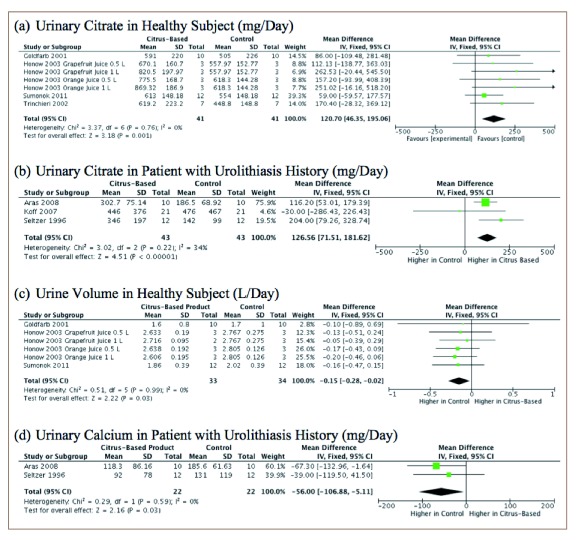
Subgroup analysis of urine profiles represented by forest plot.

Subgroup analysis showed a significantly higher urinary citrate level in both the healthy population and the population with history of urolithiasis who received citrus-based therapy compared to control. However, urine pH, which showed a statistically significant increase in urine pH compared to controls, did not demonstrate any differences in a subgroup analysis. On the other hand, urinary calcium was lower after consumption of citrus-based products compared to controls in the urolithiasis population. Furthermore, this study demonstrated that there was a lower urine volume in the healthy population after drinking citrus-based products compared to controls ( Figure 5). We did not find any differences in other urine profile variables, either in the healthy population or the population with history of urolithiasis ( Supplementary Figure 1 and Supplementary Figure 2).

We tried to conduct further analysis by excluding Aras *et al.* ^20^ from quantitative analysis, due to its different study method (RCT). We still found a significant increase in urine citrate level in both mixed population (mean difference 132.46; 95% CI 70.48-194.44) and the population with a history of urolithiasis (mean difference 159.22; 95% CI 47.05-271.40]), as well as no statistically significant difference in urine pH (mean difference 0.16; 95% CI -0.02-0.33). Furthermore, other variables still demonstrate similar outcomes after exclusion of Aras *et al*.

### Comparison between citrus-based products and potassium citrate in urine profile

Due to the reasons stated above, we decided to discuss the comparisons between citrus-based product and potassium citrate in a qualitative manner.

Three studies showed both citrus-based products (lemon juice and lime powder) and potassium citrate increased the level of urinary citrate significantly ^17, 19, 20^. Even though no significant difference in post treatment urine profile was found between citrus-based products and potassium citrate, post-treatment citrate level in the potassium citrate group showed a 3.5 times increase from pre-treatment level, while it was only 2.5 times in the lemon juice group ^20^. Furthermore, Penniston *et al*. exhibited a greater maximum increase of urinary citrate level in lemonade therapy combined with potassium citrate compared to lemonade therapy alone ^17^.

Two studies also showed significant increase in urine pH in both treatment arms ^17, 19^. However, a study by Aras *et al*. only exhibit a significant increase in urine pH for the potassium citrate group ^20^. In terms of side effects, patients in the potassium citrate group suffered gastric and oropharyngeal discomfort, although they did not require drug discontinuation ^20^. Furthermore, potassium citrate had lower compliance compared to citrus-based therapy ^19^.

Characteristics of studies included for urine pH Click here for additional data file. Copyright: © 2017 Rahman F et al. 2017 Data associated with the article are available under the terms of the Creative Commons Zero "No rights reserved" data waiver (CC0 1.0 Public domain dedication).

Characteristics of studies included for urinary citrate Click here for additional data file. Copyright: © 2017 Rahman F et al. 2017 Data associated with the article are available under the terms of the Creative Commons Zero "No rights reserved" data waiver (CC0 1.0 Public domain dedication).

Characteristics of studies included for urine volume Click here for additional data file. Copyright: © 2017 Rahman F et al. 2017 Data associated with the article are available under the terms of the Creative Commons Zero "No rights reserved" data waiver (CC0 1.0 Public domain dedication).

Characteristics of studies included for urinary calcium Click here for additional data file. Copyright: © 2017 Rahman F et al. 2017 Data associated with the article are available under the terms of the Creative Commons Zero "No rights reserved" data waiver (CC0 1.0 Public domain dedication).

Characteristics of studies included for urinary oxalate Click here for additional data file. Copyright: © 2017 Rahman F et al. 2017 Data associated with the article are available under the terms of the Creative Commons Zero "No rights reserved" data waiver (CC0 1.0 Public domain dedication).

Characteristics of studies included for urinary uric acid Click here for additional data file. Copyright: © 2017 Rahman F et al. 2017 Data associated with the article are available under the terms of the Creative Commons Zero "No rights reserved" data waiver (CC0 1.0 Public domain dedication).

## Discussion

This study showed that citrus-based products, such as lemonade, orange juice and grapefruit juice, could increase urinary citrate levels and urine pH. Low citrate excretion, as in type I tubular acidosis, shows an increase in nephrolithiasis incidence ^27^. Therefore, existence of citrate in urine is important since it is a well-known preventive factor in calcium stone formation, with an increase in calcium salt solubility and calcium oxalate crystal growth inhibition as its primary mechanism. It also can reduce bone resorption and increase calcium reabsorption in kidneys. Furthermore, citrate fixes the inhibitory properties of Tamm-Horsfall protein ^28^. Citrate and Tamm-Horsfall protein are related to inhibition of calcium oxalate agglomeration ^29^. An increase in urine pH is due to metabolism of citrate into bicarbonate ^13^. Moreover, an increase in urine pH could reduce reabsorption of citrate ^30^. Thus, it could induce more citrate excretion. A study conducted by Curhan *et al.* ^31^ found an increased risk of stone formation associated with grapefruit juice consumption; although, the exact mechanism is still unclear. One theory suggests that grapefruit juice contains sugar, which can increase calcium excretion ^31^. However, this study proved that citrus-based products could increase urinary citrate level, which could be a protective factor for urinary tract stone formation.

Potassium citrate has been used as urolithiasis stone treatment for more than two decades. Its effectiveness in urolithiasis treatment has been established from several studies ^32, 33^. From one meta-analysis conducted by Phillip *et al.*, potassium citrate significantly reduced stone size, reduced new stone formation, and increased citrate levels ^34^. The stone prevention mechanism of potassium citrate is thought to be due to alkali loading and its citrate-uric effect ^35^. In this study, potassium citrate showed a significant increase in urinary citrate and is superior to citrus-based products in elevating urinary citrate. However, the use of potassium citrate has a limitation due to its side effect if used for a long term period, such as epigastric discomfort and frequent large bowel movement, and it requires the consumption of many tablets daily to reach sufficient therapeutic doses, which could dramatically decrease patient compliance ^36^. Therefore, citrus-based products could be an alternative therapy with lower cost and better urinary citrate level than control therapy.

This is the first systematic review and meta-analysis that focuses on citrus-based product and its effect towards urine profile compared to standard therapy. However, this study only searched for published article which could lead into publication bias. Moreover, most of the included studies were not conducted using the best method for interventional studies, which is randomized controlled trials. Therefore, from the positive results this study has shown, we encourage other researchers to conduct randomized controlled trials to provide stronger evidence the beneficial effects of citrus-based products on urinary stone disease.

## Conclusions

Citrus-based products increase urinary citrate and urine pH significantly compared to control treatments. Compared to standard potassium citrate therapy, there was a smaller increase in urine pH and urine citrate using citrus-based products. However, due to potassium citrate side effects and patient’s poor compliance, citrus-based products could be alternative therapy in preventing stone formation. These study’s results should encourage further research to explore citrus-based product as a urolithiasis treatment.

## Data availability

The data referenced by this article are under copyright with the following copyright statement: Copyright: © 2017 Rahman F et al.

Data associated with the article are available under the terms of the Creative Commons Zero "No rights reserved" data waiver (CC0 1.0 Public domain dedication). 

Dataset 1: Characteristics of studies included for urine pH. doi, 10.5256/f1000research.10976.d153056 ^37^

Dataset 2: Characteristics of studies included for urinary citrate. doi, 10.5256/f1000research.10976.d153057 ^38^

Dataset 3: Characteristics of studies included for urine volume. doi, 10.5256/f1000research.10976.d153058 ^39^

Dataset 4: Characteristics of studies included for urinary calcium. doi, 10.5256/f1000research.10976.d153059 ^40^

Dataset 5: Characteristics of studies included for urinary oxalate. doi, 10.5256/f1000research.10976.d153060 ^41^

Dataset 6: Characteristics of studies included for urinary uric acid. doi, 10.5256/f1000research.10976.d153061 ^42^

## Supplementary material

Supplementary Table 1. Newcastle-Ottawa scale for retrospective study’s risk of bias assessment.

.

Supplementary Table 2: PRISMA checklist.

.

Supplementary Figure 1. Urine pH, urinary calcium, urinary oxalate, and urinary uric acid in healthy subject population.

.

Supplementary Figure 2. Urine volume, urine pH, urinary oxalate, and urinary uric acid in population with a history of urolithiasis.

## References

[1] Eknoyan G: History of urolithiasis. *Clinical Reviews in Bone and Mineral Metabolism.* 2004;2(3):177–85. 10.1385/BMM:2:3:177

[2] Trinchieri A: Epidemiology of urolithiasis. *Arch Ital Urol Androl.* 1996;68(4):203–49. 8936716

[3] Türk C Knoll T Petrik A: Pocket Guidelines on urolithiasis. *Eur Urol.* 2014;40(4):362–71. 10.1159/000049803 11713390

[4] Fukuhara H Ichiyanagi O Kakizaki H: Clinical relevance of seasonal changes in the prevalence of ureterolithiasis in the diagnosis of renal colic. *Urolithiasis.* 2016;44(6):529–537. 10.1007/s00240-016-0896-3 27314408 PMC5063892

[5] Muslumanoglu AY Binbay M Yuruk E: Updated epidemiologic study of urolithiasis in Turkey. I: Changing characteristics of urolithiasis. *Urol Res.* 2011;39(4):309–14. 10.1007/s00240-010-0346-6 21161646

[6] Edvardsson VO Indridason OS Haraldsson G: Temporal trends in the incidence of kidney stone disease. *Kidney Int.* 2013;83(1):146–52. 10.1038/ki.2012.320 22992468

[7] Scales CD Jr Smith AC Hanley JM: Prevalence of kidney stones in the United States. *Eur Urol.* 2012;62(1):160–5. 10.1016/j.eururo.2012.03.052 22498635 PMC3362665

[8] Indonesia IAU: Tatalaksana Batu Saluran Kemih.2007.

[9] Singh P Enders FT Vaughan LE: Stone Composition Among First-Time Symptomatic Kidney Stone Formers in the Community. *Mayo Clin Proc.* 2015;90(10):1356–65. 10.1016/j.mayocp.2015.07.016 26349951 PMC4593754

[10] Moses R Pais VM Jr Ursiny M: Changes in stone composition over two decades: evaluation of over 10,000 stone analyses. *Urolithiasis.* 2015;43(2):135–9. 10.1007/s00240-015-0756-6 25689875

[11] Park S Pearle MS: Pathophysiology and management of calcium stones. *Urol Clin North Am.* 2007;34(3):323–34. 10.1016/j.ucl.2007.04.009 17678983

[12] Maalouf N: Approach to the adult kidney stone former. *Clin Rev Bone Min Metab.* 2012;10(1):38–49. 10.1007/s12018-011-9111-9 22654574 PMC3361075

[13] Tracy CR Pearle MS: Update on the medical management of stone disease. *Curr Opin Urol.* 2009;19(2):200–4. 10.1097/MOU.0b013e328323a81d 19188774

[14] Siener R: Can the manipulation of urinary pH by beverages assist with the prevention of stone recurrence? *Urolithiasis.* 2016;44(1):51–6. 10.1007/s00240-015-0844-7 26614113

[15] Higgins JP Altman DG Gøtzsche PC: The Cochrane Collaboration’s tool for assessing risk of bias in randomised trials. *BMJ.* 2011;343:d5928. 10.1136/bmj.d5928 22008217 PMC3196245

[16] Wells GA Shea B O’Connell D: The Newcastle-Ottawa Scale (NOS) for assessing the quality of nonrandomised studies in meta-analyses.Ottawa, ON: Ottawa Hospital Research Institute; [Accessed September 1, 2016].2011 Reference Source

[17] Penniston KL Steele TH Nakada SY: Lemonade therapy increases urinary citrate and urine volumes in patients with recurrent calcium oxalate stone formation. *Urology.* 2007;70(5):856–60. 10.1016/j.urology.2007.06.1115 17919696

[18] Sumorok NT Asplin JR Eisner BH: Effect of diet orange soda on urinary lithogenicity. *Urol Res.* 2012;40(3):237–41. 10.1007/s00240-011-0418-2 21858427

[19] Tosukhowong P Yachantha C Sasivongsbhakdi T: Citraturic, alkalinizing and antioxidative effects of limeade-based regimen in nephrolithiasis patients. *Urol Res.* 2008;36(3–4):149–55. 10.1007/s00240-008-0141-9 18560820

[20] Aras B Kalfazade N Tuğcu V: Can lemon juice be an alternative to potassium citrate in the treatment of urinary calcium stones in patients with hypocitraturia? A prospective randomized study. *Urol Res.* 2008;36(6):313–7. 10.1007/s00240-008-0152-6 18946667

[21] Goldfarb DS Asplin JR: Effect of grapefruit juice on urinary lithogenicity. *J Urol.* 2001;166(1):263–7. 10.1016/S0022-5347(05)66142-3 11435883

[22] Hönow R Laube N Schneider A: Influence of grapefruit-, orange- and apple-juice consumption on urinary variables and risk of crystallization. *Br J Nutr.* 2003;90(2):295–300. 10.1079/BJN2003897 12908889

[23] Koff SG Paquette EL Cullen J: Comparison between lemonade and potassium citrate and impact on urine pH and 24-hour urine parameters in patients with kidney stone formation. *Urology.* 2007;69(6):1013–6. 10.1016/j.urology.2007.02.008 17572176

[24] Odvina CV: Comparative value of orange juice versus lemonade in reducing stone-forming risk. *Clin J Am Soc Nephrol.* 2006;1(6):1269–74. 10.2215/CJN.00800306 17699358

[25] Seltzer MA Low RK McDonald M: Dietary manipulation with lemonade to treat hypocitraturic calcium nephrolithiasis. *J Urol.* 1996;156(3):907–9. 10.1016/S0022-5347(01)65659-3 8709360

[26] Trinchieri A Lizzano R Bernardini P: Effect of acute load of grapefruit juice on urinary excretion of citrate and urinary risk factors for renal stone formation. *Dig Liver Dis.* 2002;34(Suppl 2):S160–3. 10.1016/S1590-8658(02)80186-4 12408462

[27] Khanniazi MK Khanam A Naqvi SA: Study of potassium citrate treatment of crystalluric nephrolithiasis. *Biomed Pharmacother.* 1993;47(1):25–8. 10.1016/0753-3322(93)90033-H 8329662

[28] Fuselier HA Ward DM Lindberg JS: Urinary Tamm-Horsfall protein increased after potassium citrate therapy in calcium stone formers. *Urology.* 1995;45(6):942–6. 10.1016/S0090-4295(99)80112-5 7771027

[29] Laube N Jansen B Hesse A: Citric acid or citrates in urine: which should we focus on in the prevention of calcium oxalate crystals and stones? *Urol Res.* 2002;30(5):336–41. 10.1007/s00240-002-0272-3 12389124

[30] Heilberg IP Goldfarb DS: Optimum nutrition for kidney stone disease. *Adv Chronic Kidney Dis.* 2013;20(2):165–74. 10.1053/j.ackd.2012.12.001 23439376

[31] Curhan GC Willett WC Rimm EB: Prospective study of beverage use and the risk of kidney stones. *Am J Epidemiol.* 1996;143(3):240–7. 10.1093/oxfordjournals.aje.a008734 8561157

[32] Robinson MR Leitao VA Haleblian GE: Impact of long-term potassium citrate therapy on urinary profiles and recurrent stone formation. *J Urol.* 2009;181(3):1145–50. 10.1016/j.juro.2008.11.014 19152932

[33] Allie-Hamdulay S Rodgers AL: Prophylactic and therapeutic properties of a sodium citrate preparation in the management of calcium oxalate urolithiasis: randomized, placebo-controlled trial. *Urol Res.* 2005;33(2):116–24. 10.1007/s00240-005-0466-6 15871014

[34] Phillips R Hanchanale VS Myatt A: Citrate salts for preventing and treating calcium containing kidney stones in adults. *Cochrane Database Syst Rev.* 2015;10(10):CD010057. 10.1002/14651858.CD010057.pub2 26439475 PMC9578669

[35] Ettinger B Pak CY Citron JT: Potassium-magnesium citrate is an effective prophylaxis against recurrent calcium oxalate nephrolithiasis. *J Urol.* 1997;158(6):2069–73. 10.1016/S0022-5347(01)68155-2 9366314

[36] Lee YH Huang WC Tsai JY: The efficacy of potassium citrate based medical prophylaxis for preventing upper urinary tract calculi: a midterm followup study. *J Urol.* 1999;161(5):1453–7. 10.1016/S0022-5347(05)68925-2 10210371

[37] Rahman F Birowo P Widyahening IS: Dataset 1 in: Effect of citrus-based products on urine profile: A systematic review and meta-analysis. *F1000Research.* 2017 Data Source 10.12688/f1000research.10976.1 PMC5428529 28529700

[38] Rahman F Birowo P Widyahening IS: Dataset 2 in: Effect of citrus-based products on urine profile: A systematic review and meta-analysis. *F1000Research.* 2017 Data Source 10.12688/f1000research.10976.1 PMC5428529 28529700

[39] Rahman F Birowo P Widyahening IS: Dataset 3 in: Effect of citrus-based products on urine profile: A systematic review and meta-analysis. *F1000Research.* 2017 Data Source 10.12688/f1000research.10976.1 PMC5428529 28529700

[40] Rahman F Birowo P Widyahening IS: Dataset 4 in: Effect of citrus-based products on urine profile: A systematic review and meta-analysis. *F1000Research.* 2017 Data Source 10.12688/f1000research.10976.1 PMC5428529 28529700

[41] Rahman F Birowo P Widyahening IS: Dataset 5 in: Effect of citrus-based products on urine profile: A systematic review and meta-analysis. *F1000Research.* 2017 Data Source 10.12688/f1000research.10976.1 PMC5428529 28529700

[42] Rahman F Birowo P Widyahening IS: Dataset 6 in: Effect of citrus-based products on urine profile: A systematic review and meta-analysis. *F1000Research.* 2017 Data Source 10.12688/f1000research.10976.1 PMC5428529 28529700

