# Supplementary material for: Effect of citrus-based products on urine profile: A systematic review and meta-analysis
Source: F1000Res. 2017 Mar 6;6:220. [Version 1] doi: 10.12688/f1000research.10976.1 (PMC5428529; doi:10.12688/f1000research.10976.1)
Supplement: Supplementary file 7 [file f1000research-6-11834-s0006.tgz › 3e326d6c-a2cf-4e19-9b0d-e6257489985b.docx]

| No. | Criteria | Description |
| --- | --- | --- |
| *Selection* | | |
| 1. | Representativeness of the exposed cohort | Somewhat representative. Patients were adults with calcium oxalate stone formation and had three or more visits to Metabolic Stone Clinic and three or more Urorisk Diagnostic profiles. Subjects in exposed cohorts were using lemonade (group cohort). |
| 2. | Selection of the non-exposed cohort | Subjects in non-exposed cohort were drawn from the same community as the exposed cohort. Subjects in non-exposed cohort were using lemonade therapy and potassium citrate. |
| 3. | Ascertainment of exposure | Ascertainment of exposure were retrieved from medical records. |
| 4. | Demonstration that outcome of interest was not present at start of study | Outcome was urine profile after. |
| *Comparability* | | |
| 1. | Comparability of cohorts on the basis of the design or analysis | Study controls for its diet. |
| *Outcome* | | |
| 1. | Assessment of outcome | Confirmation of the outcome came from secure records |
| 2. | Was follow-up long enough for outcomes to occur | This is retrospective cohort. However, the follow up was enough |
| 3. | Adequacy of follow up of cohorts | All the data were available. |

**Supplementary Table 1: Newcastle-Ottawa scale for retrospective study’s risk of bias assessment.**
