# Supplementary figures and images for: Effect of citrus-based products on urine profile: A systematic review and meta-analysis
Source: F1000Res. 2017 Mar 6;6:220. [Version 1] doi: 10.12688/f1000research.10976.1 (PMC5428529; doi:10.12688/f1000research.10976.1)

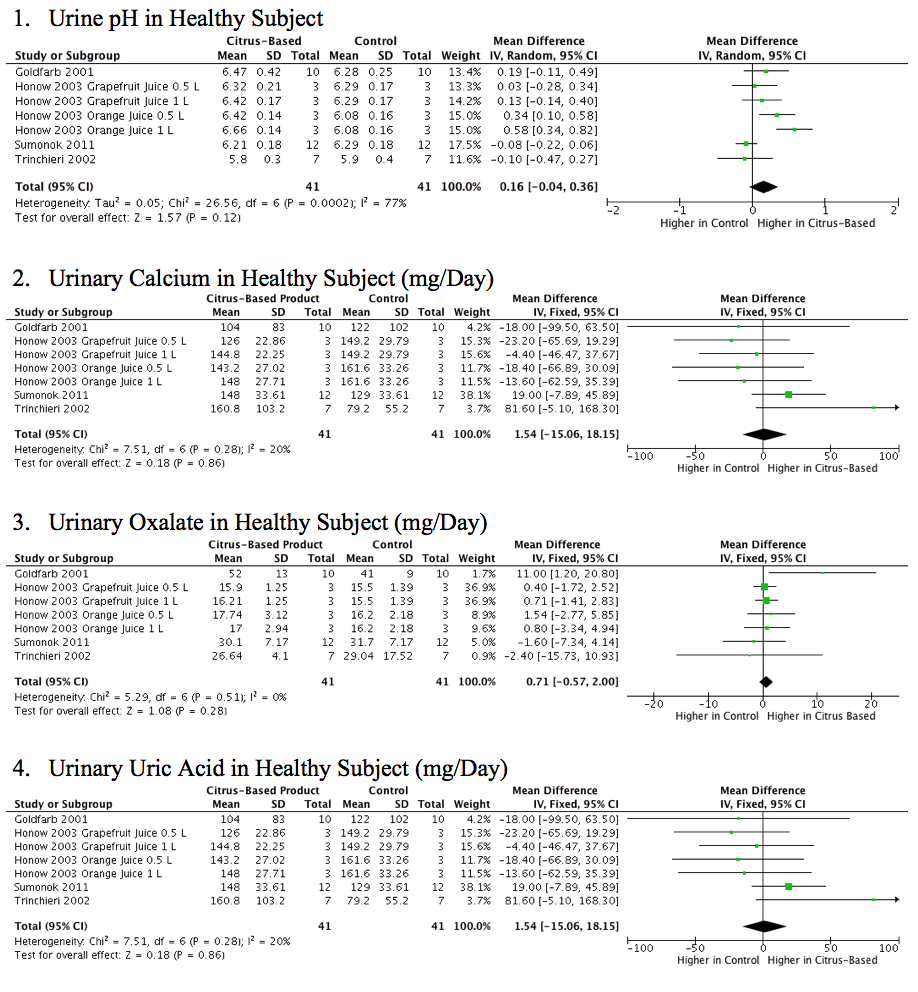

Supplement: Supplementary file 9 [file f1000research-6-11834-s0008.tgz › 1dba806e-eafe-4d13-8ce9-a5b094bb45d4.png]

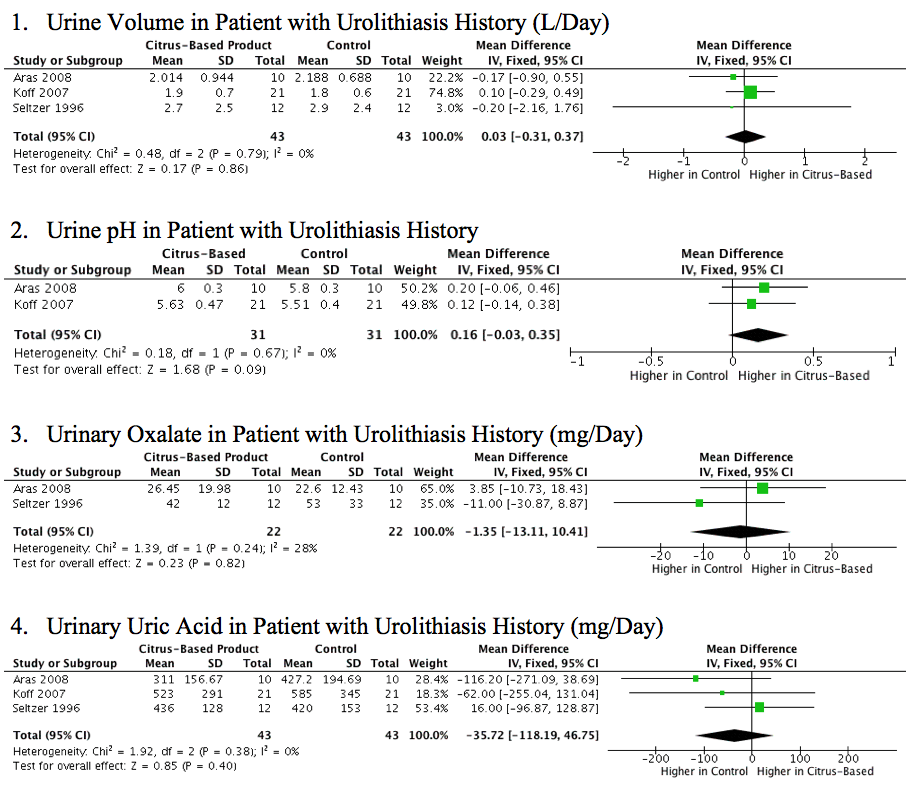

Supplement: Supplementary file 10 [file f1000research-6-11834-s0009.tgz › 50d3ad3f-c773-4597-8698-246322a918b4.png]
