# Supplementary material for: Effect of citrus-based products on urine profile: A systematic review and meta-analysis
Source: F1000Res. 2017 Mar 6;6:220. [Version 1] doi: 10.12688/f1000research.10976.1 (PMC5428529; doi:10.12688/f1000research.10976.1)
Supplement: Peer review history

# Peer review: Referee response for version 1

**DOI:** 10.5256/f1000research.11834.r22426

**Published:** 2017-05-08

**Authors:** Bhaskar K Somani

**Affiliations:** 1 University Hospitals Southampton NHS Trust, Urology, Southampton, SO16 6YD., UK

The authors present a comprehensive systematic review and meta-analysis of the effect of citrus-based products on urine profile. The paper is supplemented by the PRISMA flow chart and forest plot charts to present their results.

 Although their search strategy is good, they should have used other terms such as 'kidney stones', 'stones', 'ureteric stones' and 'calculi' too. They have only used the term 'Urolithiasis' which could potentially miss on other relevant studies.

 As a limitation of any systematic review the authors are correct in acknowledging that their is likely to be a publication bias. Similarly, the long term effect of the use of citrate-based products in not know from this study and whether their results translate into a reduction in stone recurrences remain unknown. 

 A recent cochrane review on the use of citrate salts on prevention and treatment of calcium containing kidney stones in adults showed a reduction in new stone formation and stone recurrences in these patients ^1^.

 Overall the paper reads well and is a nice summary on the use of citrate based products on urine profile.

I have read this submission. I believe that I have an appropriate level of expertise to confirm that it is of an acceptable scientific standard.

## References

[1] PhillipsRHanchanaleVSMyattASomaniBNabiGBiyaniCS: Citrate salts for preventing and treating calcium containing kidney stones in adults. *Cochrane Database Syst Rev*.2015; 10.1002/14651858.CD010057.pub2 CD010057 10.1002/14651858.CD010057.pub2 26439475PMC9578669

# Peer review: Referee response for version 1

**DOI:** 10.5256/f1000research.11834.r21950

**Published:** 2017-05-02

**Authors:** David S Goldfarb

**Affiliations:** 1 NYU Langone Medical Center and NYU School of Medicine, New York, NY, USA

1. The authors are correct in stating that no previous meta-analysis of the effects of citrus fruits has been performed. The results are not surprising as stone clinicians consider citrus supplementation (or potassium citrate) of important utility. However, the meta-analysis is reasonable to perform as the intervention is commonly administered.

 2.The studies are similar enough to consider a meta-analysis worth doing. One study used diet orange soda which is not citrus juice (I am the senior author of that study). There is otherwise an appropriate selection of studies, based on availability of the requisite data which the authors explain in detail.  

 3. The limitations of the data include the relatively small sample size, so that the meta-analysis is also underpowered but shows the expected increase in urine citrate and the increase in urine pH only in stone formers and not in non-stone forming controls. No studies actually assess stone formation as an outcome, addressing only urinary chemistry. 

 4. The conclusions appear reasonable, and are well-stated, if not surprising. 

 5. Table 1 could be improved by including the dose of juice for all the interventions.

 6. I did not find a legend for figure 2 about bias assessment. The figure is hazy, not of perfect resolution. There is no interpretation of the bias assessment in the manuscript. It is worth noting that it is probably not possible to blind participants to citrus juice vs water. The other criteria, such as blinding to sequence allocation may also not be critical to a study of urine chemistry and not of kidney stone outcomes. 

 7. Regarding Curhan’s finding that grapefruit juice was associated with higher risk for stones, mentioned in the discussion, that finding was not confirmed in a later study ^1^.

 8. In the introduction, the authors state “whereas citrate could prevent stone formation by ionizing urinary calcium”: this is not quite correct. Citrate binds to ionic calcium and prevents it from binding to oxalate or phosphate.

I have read this submission. I believe that I have an appropriate level of expertise to confirm that it is of an acceptable scientific standard.

## References

[1] FerraroPMTaylorENGambaroGCurhanGC: Soda and other beverages and the risk of kidney stones. *Clin J Am Soc Nephrol*.2013;8(8) : 10.2215/CJN.11661112 1389-95 10.2215/CJN.11661112 23676355PMC3731916
